# Supplementary material for: Quantitative Analysis of the Trade-Offs of Colony Formation for Trichodesmium
Source: Microbiol Spectr. 2022 Nov 14;10(6):e02025-22. doi: 10.1128/spectrum.02025-22 (PMC9769814; doi:10.1128/spectrum.02025-22)
Supplement: Supplemental file 1 — Fig. S1. Download spectrum.02025-22-s0001.pdf, PDF file, 0.09 MB [file spectrum.02025-22-s0001.pdf]

Supplemental material

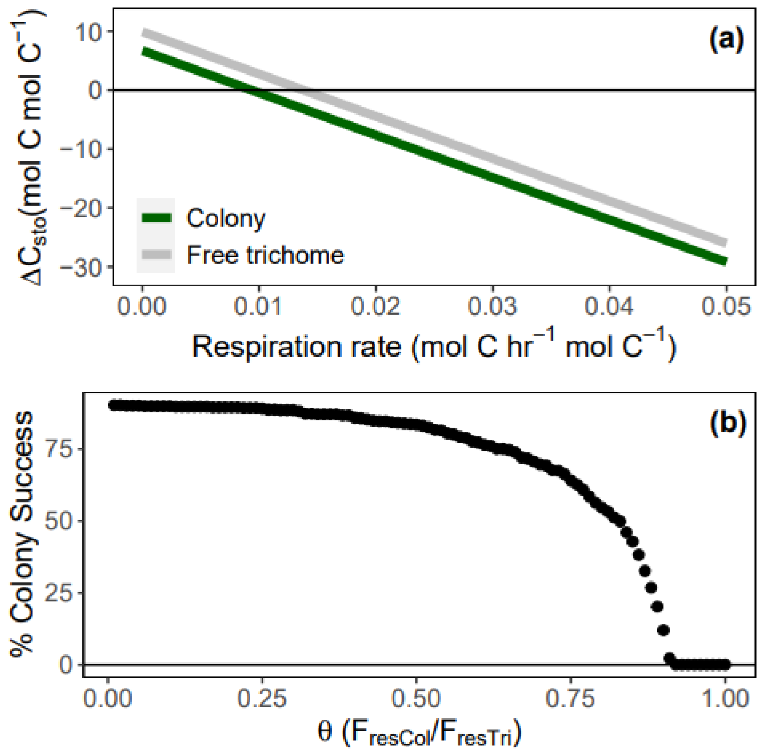

Figure S1: (A) Change in total carbon storage $(mol\ C\ mol\ C^{-1})$ for both colonies and free trichomes at the end of a 30-day model simulation with fixed and equal respiration costs (B) Proportion of simulations where colonies outcompete free trichomes as θ is varied. θ is the ratio of the respiration rate of colonies and the respiration rate of free trichomes. Green refers to colonies and grey refers to the free trichomes.
